# New Insights into the Significance of PARP-1 Activation: Flow Cytometric Detection of Poly(ADP-Ribose) as a Marker of Bovine Intramammary Infection

**DOI:** 10.3390/cells10030599

**Published:** 2021-03-09

**Authors:** Giovanna De Matteis, Francesco Grandoni, Michele Zampieri, Anna Reale, Maria Carmela Scatà

**Affiliations:** 1 Research Centre for Animal Production and Aquaculture, Consiglio per la Ricerca in Agricoltura e l’Analisi dell’Economia Agraria (CREA), Via Salaria, 31-00015-Monterotondo, 7-00161 Rome, Italy; francesco.grandoni@crea.gov.it (F.G.); mariacarmela.scata@crea.gov.it (M.C.S.); 2 Department of Experimental Medicine, Faculty of Medicine and Dentistry, Sapienza University of Rome, Via Chieti, 7-00161 Rome, Italy; michele.zampieri@uniroma1.it (M.Z.); anna.reale@uniroma1.it (A.R.)

**Keywords:** bovine mastitis, flow cytometry, poly(ADP-ribose), PAR, PARP-1, active Caspase-3

## Abstract

Bovine intramammary infections are common diseases affecting dairy cattle worldwide and represent a major focus of veterinary research due to financial losses and food safety concerns. The identification of new biomarkers of intramammary infection, useful for monitoring the health of dairy cows and wellness verification, represents a key advancement having potential beneficial effects on public health. In vitro experiments using bovine peripheral blood mononuclear cells (PBMC), stimulated with the bacterial endotoxin lipopolysaccharide (LPS) enabled a flow cytometric assay in order to evaluate in vivo poly-ADP-ribose (PAR) levels. Results showed a significant increase of PAR after 1 h of treatment, which is consistent with the involvement of PARP activity in the inflammatory response. This study investigated PARP-1 activation in leukocyte subpopulations from bovine milk samples during udder infection. A flow cytometric assay was, therefore, performed to evaluate the PAR content in milk leukocyte subsets of cows with and without intramammary infection (IMI). Results showed that milk lymphocytes and macrophages isolated from cows with IMI had a significant increase of PAR content compared to uninfected samples. These results suggest mastitis as a new model for the study of the role of PARP in zoonotic inflammatory diseases, opening a new perspective to the “One Health” approach.

## 1. Introduction

The global health of humans and animals is interconnected, indicating the need for a “One Health” perspective. Zoonoses, infectious diseases that are naturally transmitted from animals to humans, and antimicrobial resistance are recognized as threats to global health and pose a major public health concern. Food is one of the most important vehicles for the transmission of resistant bacteria from animals to humans, and the consumption of food containing antibiotic-resistant bacteria has led to the acquisition of antimicrobial-resistant infections. Therefore, animal welfare and antibiotic resistance are now important issues for consumers and for the expectations of citizens regarding the livestock sector [1].

Mastitis, the inflammation of the mammary gland, is a major disease affecting dairy cattle worldwide and is one of the major topics in veterinary research due to financial losses and food safety concerns over antimicrobial use.

Somatic cell count (SCC) has been used for a long time as an indicator of udder health; however, this parameter is not always a clear indicator of a potential infection. In addition, SSC does not distinguish between the principal cell populations present in milk, including lymphocytes, neutrophils, and macrophages, which play an important role in the defense of mammary gland against invading pathogens and in the resolution of infectious disease [2].

In recent years, new approaches for the diagnosis of mastitis, such as the Differential Somatic Cell Count (DSCC), have been proposed [3]; however, with this approach, it is not yet possible to characterise the subclinical forms.

For these reasons, the development of new markers and technologies to prevent, diagnose and treat intramammary infections is increasingly important to ensure a safe and healthy product.

Inflammation is a fundamental biological process that underlies many acute and chronic pathological conditions. It occurs in response to any alteration of tissue integrity in order to restore tissue homeostasis through the induction of various repair mechanisms. It is known that the excessive production of reactive oxidant species promotes the development of a state of oxidative stress and may have significant biological consequences such as the development and perpetuation of inflammation [4]. Furthermore, oxidative stress can cause DNA breaks, which induce activation of the poly(ADP-ribose) polymerase-1 (PARP-1), a ubiquitous nuclear enzyme. Activated PARP-1 synthesises poly(ADP-ribose) (PAR) using the nicotinamide adenine dinucleotide oxidized form (NAD^+^) as a substrate [5,6].

In recent years, several studies demonstrated that PARP-1 plays a role in inflammation by influencing multiple key events such as the production of cytokines and the expression of adhesion molecules via its interaction with various pro-inflammatory transcription factors [7]. NF-κB is one of the best known pro-inflammatory transcription factors that are positively regulated by PARP-1 during inflammatory responses induced by various stimuli, including oxidative stress, bacterial products (LPS), and inflammatory cytokines (IL-1, TNFα) [8].

Research on the involvement of PARP-1 in bovine intramammary infection is limited. We have recently reported that unhealthy udders with high SCC show changes in milk somatic cell composition and increased intracellular poly(ADP-ribose) levels [9]. More recently, we showed that the conventional flow cytometry analysis applied to milk samples is a useful tool to detect new immunological markers as potential indicators of udder health [10].

Based on this premise, with a view to exploiting PARP activity as a new potential marker of udder health, we have evaluated the PAR level in milk leukocyte subpopulations during udder infection.

## 2. Materials and Methods

### 2.1. Samples Collection

In the study, 19 multiparous Italian Holstein lactating cows were enrolled. The animals were kept at the Research Centre for Animal Production and Aquaculture of CREA. The management and care of the animals were carried out in compliance with the 2010/63/EU directive and the Italian regulation D. Lgs n. 26/2014 and under ethical approval and experimental license from the Health Ministry authorization n. 529/2017-PR.

The milk and blood samples from cows were collected according to good veterinary practice.

Composite milk samples were collected during routine management controls around 2 weeks post-calving. Single quarter samples were collected in sterile tubes by hand milking after accurate cleaning and disinfection of the udder and discarding of the 1st streaks of milk. The samples were collected by taking an equal amount from all 4 quarters. Aliquots of milk samples (50 mL) were delivered refrigerated on the same day to an external laboratory (Istituto Zooprofilattico Sperimentale Lazio e Toscana, IZSLT, Rome, Italy) for bacteriological analysis. This test was performed by traditional bacterial culture for the identification of bovine mastitis bacteria.

The Somatic Cell Count (SCC) was performed by a DeLaval Cell counter instrument (DeLaval International AB, Tumba, Sweden).

### 2.2. PAR Detection in LPS Stimulated Bovine Peripheral Blood Mononuclear Cells (PBMC)

For “in vitro” experiments, jugular vein peripheral blood samples (10 mL) were collected from 3 healthy lactating cows into vacutainer tubes containing K_3_-EDTA (Beckton Dickinson, Plymouth, UK). PBMC were purified as previously reported by De Matteis and co-workers [6].

To verify PARP activation upon inflammatory stimulation, PBMC were treated in vitro with the bacterial endotoxin lipopolysaccharide (LPS, *E. coli*, O55:B5) (Sigma-Aldrich MO, USA). Aliquots of PBMC (2 × 10^6^ cells/mL) were resuspended in RPMI-1640 medium supplemented with 10% of fetal bovine serum and incubated in 24 well plates at 37 °C and 5% of CO_2_ for 30 min, to restore resting conditions before the addition of LPS (Sigma-Aldrich) at a final concentration of 1 µg/mL. To compare the dynamic response of PARP activity upon short or long incubation times, LPS treatment was carried out for 1 h and 16 h. A positive control of PAR detection specificity was carried out treating cells with LPS at 1 h in the presence or absence of ABT-888 (Veliparib) (MedChemExpress LLC, Monmouth Junction, NJ, USA) at a final concentration of 1 μM). Alternatively, cells were treated with either doxorubicin (Sigma-Aldrich, final concentration 1 μM) or ABT-888, and with a combination of both drugs for 2 h. Cells were then washed twice with Phosphate Buffered Saline solution (PBS), followed by treatment with the Cytofix/Cytoperm Fixation/Permeabilisation Solution Kit (BD Biosciences, San Jose, CA, USA).

Preliminary tests were carried out to adapt the fixation/permeabilisation Solution Kit (BD Biosciences) protocol to bovine cell analysis. Furthermore, in order to exclude that the increasing of PAR levels detected by flow cytometry was due to the fixation/permeabilisation conditions, various incubation times with the permeabilising solution were tested. An optimal incubation time for fixation/permeabilisation step was established at 20 min (data not shown).

In order to detect the intracellular PAR levels [11], the cells were then incubated with saturating amounts of the mouse anti-PAR antibody (clone 10H ALX-804-220, Enzo Life Sciences, Inc. Farmingdale, NY, USA) for 45 min at 4 °C. A Goat anti-mouse IgG-polyclonal antibody AlexaFluor 488-conjugated was used as a secondary antibody (Thermo Fisher Scientific, Waltham, MA, USA). Purified mouse anti-KLH antibody was used as IgG3 isotype control (Trevigen, Gaithersburg, MD, USA).

In order to verify apoptotic inactivation of PARP after prolonged LPS stimulation, we evaluated the activation of Caspase-3 and the cleavage of PARP-1 by using the PE- conjugated anti-active Caspase-3 (clone C92-605—Becton Dickinson) and the FITC- conjugated anti-cleaved PARP-1 (Asp214) (clone F21-852, BD—Becton Dickinson) monoclonal antibodies, respectively.

Stained cells were analysed by the CytoFlex flow cytometer and data were analyzed with the Kaluza Analysis Software 2.1 (Beckman Coulter, Brea, CA, USA). Intracellular levels were reported as PAR Mean Fluorescent Intensity (MFI) and percentage of cells positive to active Caspase-3 or cleaved PARP1.

### 2.3. Flow Cytometric Assessment of PAR in Milk Leukocytes

Aliquots (50 mL) of composite milk samples from each of all 19 cows were used for somatic cell purification. Immediately after collection, milk samples were centrifuged at 800× *g* for 20 min at 8 °C, and the fat layer and supernatant were discarded. Cell pellets were washed twice with cold PBS. Cells were then counted with a TC10^TM^ automated cell counter (BioRad Laboratories, Hercules, CA, USA) and labeled for flow cytometric analysis.

The experimental design assigned milk samples according to the results of the bacteriological analysis as naturally infected (*n* = 10) or not-infected (*n* = 9) cows.

To detect the intracellular PAR levels in milk leukocyte subpopulations, a three-colour flow cytometric assay was performed. First, the isolated cells (~1 × 10^6^ cells/100 µL) were stained with saturating quantities of the pan-leukocytes anti-CD45 PE-conjugated (clone CC1, Bio-Rad) and anti-CD14 Per-CP conjugated (TÜK4, Thermo Fisher) antibodies for 30 min at 4 °C. Dead cells were excluded by LIVE/DEAD™ Fixable Near-IR Dead Cell Staining (Thermo Fisher) performed in non-permeabilised cells. Epithelial cells were excluded as CD45 negative cells. After then, the Cytofix/Cytoperm Fixation/Permeabilisation Solution Kit (BD Biosciences) was used to detect PAR, as described above.

The flow cytometry analysis of PAR amount in milk cells was performed on a CytoFlex instrument, and data were analyzed with the Kaluza Analysis Software 2.1 (Beckman Coulter).

### 2.4. Preparation of Whole Cell Extracts and Western Blot Analysis

PBS-washed PBMC and milk somatic cells were lysed in RIPA buffer (50 mM Tris−HCl at pH 7.4, 150 mM NaCl, 1% NP-40, 0.5% sodium deoxycholate, 0.1% SDS, and 1 mM EDTA), supplemented protease inhibitors (SIGMAFAST Protease Inhibitor Cocktail, EDTA-Free, Merck Life Science, Darmstadt, Germany). Protein concentration was determined by the Bradford protein assay reagent (Bio-Rad Laboratories), using purified bovine serum albumin (Promega, Madison, WI, USA) as a standard for quantitation. Equal protein amounts (50 μg) were subjected to discontinuous SDS—poly-acrylamide gel electrophoresis and blotted onto the Amersham Protran nitrocellulose membrane (Merck Life Science, Darmstadt, Germany). The membranes were then treated with blocking buffer (PBS with 0.05% TWEEN 20 and 5% non-fat dry milk) followed by incubation with specific antibodies in the blocking buffer. The following primary antibodies were used: Mouse monoclonal anti-PAR (Trevigen, Gaithersburg, MD, USA), mouse monoclonal anti-PARP1 antibody (clone C2-10, Enzo Life Sciences, Farmingdale, NY, USA), mouse monoclonal anti-β-Actin (Merck Life Science), and rabbit polyclonal anti-cleaved Caspase 3 (Asp175) (Cell Signaling Technology, Danvers, MA, USA) IgGs. Secondary antibodies were either goat anti-mouse (Santa Cruz Biotechnology, Dallas, Texas, USA) IgGs or goat anti-rabbit (Trevigen), labeled with Horseradish Peroxidase (HRP). Chemiluminescence was determined using the Pierce ECL Plus Western Blotting Substrate (Thermo Fisher Scientific) and detected using the Amersham Hyperfilm ECL (Cytiva, Marlborough, MA, USA). Images were captured on the GS-800 Calibrated Densitometer (Bio-Rad).

### 2.5. Statistical Analysis

Data were given as mean ± SD. Two-group comparisons were performed by the paired or unpaired Student’s *t*-test. The one-way analysis of variance (ANOVA) was used for comparisons of more than two groups. In the case of a statistically significant ANOVA, the Bonferroni pairwise comparison test was used to determine statistically significant group differences. Association between variables was investigated by the Pearson’s r correlation coefficient. *p* values ≤0.05 were considered statistically significant.

## 3. Results

### 3.1. Flow Cytometry Detection of PAR in “In Vitro” LPS-Stimulated PBMC

The flow cytometric assay was performed to evaluate PAR level variations after short (1 h) and prolonged LPS treatment (16 h) on fresh PBMC.

**Figure 1:**
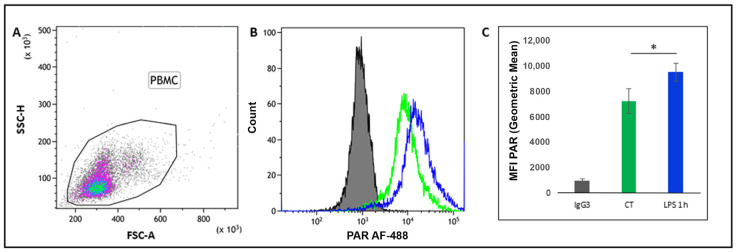
Increasing poly-ADP-ribose (PAR) content in bovine peripheral blood mononuclear cells (PBMC) in response to 1 h lipopolysaccharide (LPS) treatment. (**A**) Representative flow cytometry dot plot showing permeabilised bovine PBMC; (**B**) representative histogram overlay of anti-IgG3 Isotype Control (gray histogram), control (green line), and LPS treated samples (blue line) incubated at 37 °C for 1 h. Histograms show the amount of fluorescence labelling in the cells; (**C**) increases in mean fluorescent intensity (MFI) value (geometric mean) of PAR content in PBMC, in response to LPS (*p* = 0.031, paired Student’s *t*-test). * Significant differences with *p* < 0.05.

Flow cytometry analysis efficiently detected an increase in PAR content in total bovine PBMC treated with LPS compared to the untreated controls (Figure 1A,B). The PAR content (MFI value = Geometric Mean) was significantly increased in response to 1 h of LPS treatment (*p* = 0.031) (Figure 1C).

To verify the specificity of cytometric PAR signal, the cells were treated with ABT-888, a potent inhibitor of PARP enzymes, both with and without LPS. As shown in Figure S1A, the increase in PAR detected by flow cytometric analysis following 1 h treatment with LPS was effectively attenuated by the co-treatment with ABT-888. PAR variation was further assessed by analysing the PAR content in cell extracts, using Western blotting (Figure S1B).

In addition, cells were treated with doxorubicin (DOXO) 1 μM, a DNA damaging agent triggering PARP activation, ABT-888 1 μM, and a combination of both drugs for 2 h (Figure S1C). At this treatment time, DOXO produced non-apoptotic damage, as previously shown [12]. In addition, the change in the flow cytometric signal of PAR was consistent with the result of the Western blotting analysis, showing that the DOXO-induced PAR levels were attenuated by ABT-888 (Figure S1C,D).

**Figure 2:**
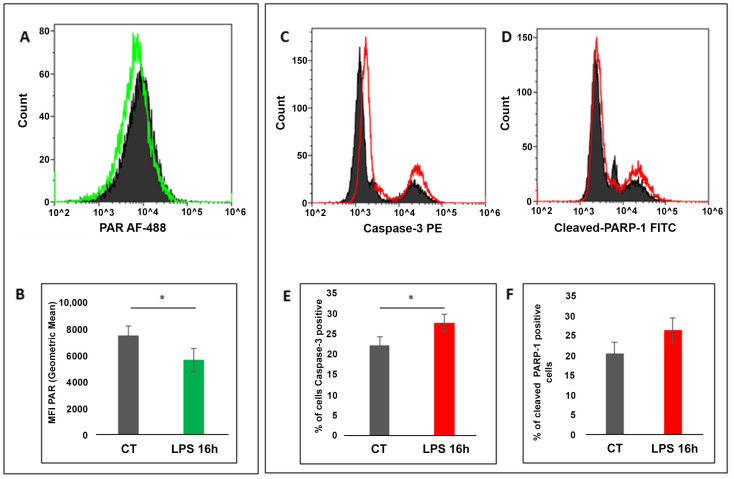
Response to 16 h LPS treatment of bovine PBMC. (**A**) Representative histograms overlay of PAR content in control (black line) and LPS treated PBMC (green line). (**B**) Histogram showing the decrease in MFI PAR (Geometric mean) in PBMC in response to 16h LPS treatment (*p* = 0.048). (**C**) Histogram overlay showing the amount of active caspase-3 positive PBMC; (**D**) histogram overlay showing the amount of cleaved PARP-1 positive PBMC. (**E**) Percentage of active Caspase-3 positive cells in control and LPS treated PBMC. (**F**) Percentage of cleaved-PARP-1 positive cells in control and LPS treated PBMC. Results are presented as mean ± SD of *n* = 3 cows. * Significant differences with *p* < 0.05, paired Student’s *t*-test.

In contrast, the 16 h LPS treatment caused a decrease in PAR levels (*p* = 0.048), suggesting reduced PARP activity (Figure 2A,B).

To investigate whether this decrement in PARP activity was due to apoptosis, we evaluated Caspase-3 activation (Figure 2C) and PARP-1 cleavage (Figure 2D) by flow cytometry.

Interestingly, results showed that the prolonged LPS stimulation caused a significant increase in the percentage of cells positive to active Caspase-3 (*p* = 0.034) and cleaved-PARP-1 (*p* = 0.074) (Figure 2E,F), which confirmed the induction of an apoptotic process.

In keeping with flow cytometry results, Western blotting analysis confirmed a reduced PAR level, the presence of cleaved PARP-1 and higher amounts of Caspase-3 cleaved fragments in cells treated with LPS for 16 h with respect to control cells (Figure S2).

### 3.2. Bacteriological Milk Analysis

The bacteriological analysis reported the presence of bacteria in 10 of the 19 milk samples. The following pathogens were detected in infected samples: *Coagulase-negative staphylococci* (*n* = 4); *Staphylococcus aureus* (*n* = 3); *Serratia marcescens* (*n* = 1), *Escherichia coli* (*n* = 1); *Streptococcus* spp. (*n* = 1).

Descriptive statistics for SCC and differences between infected (I) and Not Infected (NI) milk samples are shown in Table 1.

### 3.3. Flow Cytometry Detection of PAR Levels in Milk Leukocyte Subsets of Healthy and Infected Cows

**Figure 3:**
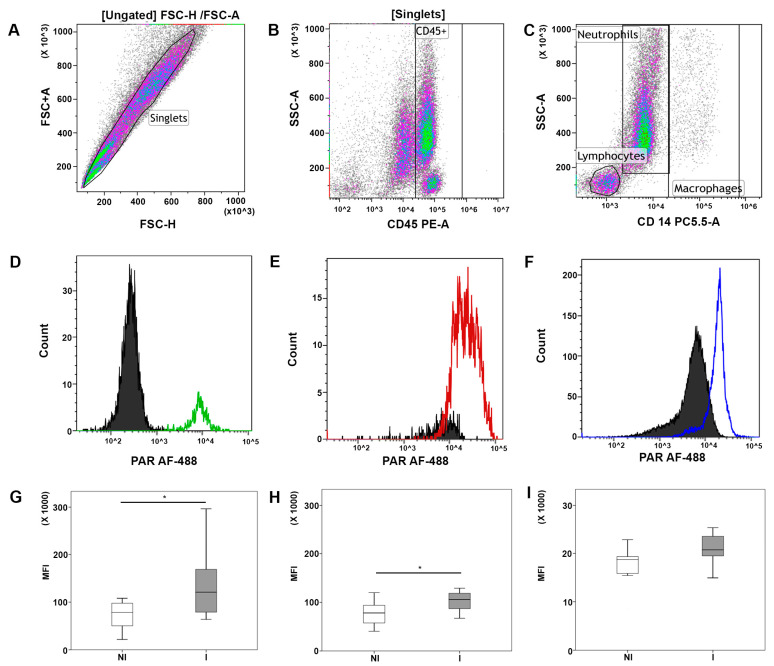
Flow cytometric evaluation of PAR in bovine milk leukocytes and PARP-1 activation in milk cells during infection. At the top, representative flow cytometry dot plots showing the gating strategy for the identification of leukocyte subpopulations: (**A**) Identification of single cells on FSC-H vs. FSC-A dot-plot, (**B**) identification of milk leukocytes (CD45^+^) in SSC vs. PE dot-plot, (**C**) identification of lymphocytes (CD14^−^) neutrophils (CD14 ^low/−^) and macrophages (CD14^+^) in SSC vs. Per-CP dot-plot. In the middle, histograms overlay showing PAR levels of the three different cell populations: (**D**) Lymphocytes, (**E**) macrophages, and (**F**) neutrophils. Below, box-whisker plots (median, 25th–75th percentiles, maximum and minimum values) showing the PAR MFI differences of the leukocyte subpopulations between not infected (NI, *n* = 9) and infected (I, *n* = 10) milk samples (**G**), lymphocytes; (**H**), macrophages, (**I**), neutrophils). A significant increase in PAR levels was observed in lymphocytes (*p* = 0.02) and macrophages (*p* < 0.01) in infected conditions after the unpaired Student’s *t*-test. * Significant differences with *p* < 0.05.

A specific gating strategy was applied to identify the leukocyte subsets of milk cells (Figure 3A–C). In order to exclude other cell types (such as epithelial cells) and debris, milk somatic cells were stained with an anti-CD45 antibody (a pan leukocytes marker) and selected by gating (Figure 3B). Dead cells were identified early with LIVE/DEAD™ Cell Staining in non-permeabilised cells, and then we marked the gate where the dead cells fell (into the gate CD45^low/neg^) and excluded them from the analysis.

Amongs milk leukocytes, the lymphocytes were defined by their typical Forward Scatter (FSC) and Side Scatter (SSC) properties and as CD45^+^/CD14^−^ cells, macrophages as CD45^+^/CD14^+^ cells while neutrophils were defined as CD45^+^/CD14^low/−^ cells (Figure 3C).

On the PAR histograms, lymphocytes, macrophages, and neutrophils were set as gates (Figure 3D–F). For each leukocyte subset, isotype control-stained cells served as a negative control. Results of flow cytometry analysis were expressed as PAR MFI, a parameter proportional to the intracellular PAR level (Figure 3G).

After setting up the flow cytometry assay for the detection of PAR in milk leukocyte subsets, we used this approach to detect the presence of udder infections.

The PAR content (MFI) was significantly higher in lymphocytes (*p* = 0.02) and macrophages (*p* < 0.01) from infected milk (I) compared to not infected samples (NI) (Figure 3G,H). In contrast, low levels of PAR and no differences between the two groups of samples were found in neutrophils (Figure 3I).

Furthermore, a linear correlation analysis revealed a positive association of SCC with the PAR content in macrophages (r = 0.464, *p* = 0.045), a tendency to a significant correlation with PAR in lymphocytes (r = 0.426, *p* = 0.060), while no significant association was found with the PAR content in neutrophils. Moreover, PAR contents in lymphocytes and macrophages were positively correlated (r = 0.523, *p* = 0.022), (Table 2).

The increased level of PAR in cells isolated from the milk of infected animals was confirmed by Western blotting experiments (Figure S3).

## 4. Discussion

Several studies in human and animal models have verified the role of PARP activity in inflammatory diseases. PARP-1 acts as a transcriptional co-regulator at the promoters of a sub-set of NF-κB-dependent pro-inflammatory genes [13]. Widdison and co-workers [14] found the up-regulation of PARP-1 and of several genes belonging to the NF-κB pathway in bovine alveolar macrophages infected with *Mycobacterium bovis*. Moreover, NF-κB has been reported to play a role in mastitis pathogenesis and its activation has been linked with mastitis [15,16].

In dairy mammals, the udder is one of the organs most exposed to functional “stress”. Mastitis causes significant economic losses in livestock species, and the major cause of mastitis is IMI caused by several pathogens. Dairy cows that suffer from IMI show a decrease in milk production, sometimes to a considerable extent. In animal husbandry, it is, therefore, of fundamental importance that an accurate and early diagnosis is made of the onset of the disease. The diagnosis of IMI is mainly based on bacteriological analysis and the count of somatic cell (SCC) in milk. The SCC measures the content of cells present in milk, indiscriminately including the immune (lymphocytes, macrophages, neutrophils) and epithelial cells. The SCC is increased and the composition in leukocyte cells changes significantly during udder inflammation [17].

In a previous study, we showed that increased SCC is associated with PARP-1 activation during bovine mastitis. In particular, we found high levels of PAR in udder quarters with high SCC. Moreover, the characterization of milk cell populations showed that quarters with high SCC exhibit an increase in phagocytes (neutrophils and macrophages). Considering that macrophages are important players in the inflammatory response, we proposed that the high PAR level found in milk with high SCC could be connected to PARP-1 activation in the increased proportion of the macrophages population [9]. According to these findings, PAR could be considered as an inflammatory mediator produced by both lymphocytes and monocytes/macrophages.

In this paper, we showed that the intracellular accumulation of PAR in specific immune cells worked as a potential marker useful to detect infected Holstein-Friesen cows with SCC lower than 300,000 cells/mL.

Flow cytometry is a technique already applied to detect basal intracellular PAR in cell lines, humans and rats and also to evaluate changes in PARP-1 activity in response to several stress stimuli [11,18,19,20,21].

The flow cytometry PAR assay on cultured bovine PBMC upon LPS treatment allowed us to set up the optimal conditions to evaluate PARP activation rapidly on a single population level.

Our in vitro results showed an increase in PAR levels after 1 h of treatment and a slight decrease after 16 h.

LPS at 1 h may induce cell damage to which the cell responds by activating PARP-1, suggesting that the damage produced is repairable. This is in line with previous studies in murine and human cell lines [22], highlighting that after LPS stimulation, the maximum level of PAR signal, measured by Western Blot analysis with the anti-PAR monoclonal antibody (Clone 10H), was reached at 1 h.

Conversely, prolonged in vitro stimulation (16 h) may induce apoptosis, cleavage of PARP-1 enzyme and, consequently, a decrease in its activity, leading to lower intracellular PAR content. The presence of signs of apoptosis in the controls could be due to the normal susceptibility of PBMCs to prolonged standard in vitro cultivation.

In fact, a pathway for controlling PARP-1 activity is represented by apoptosis, which limits the consumption of NAD^+^ by inactivating PARP-1 [23]. This process takes place through a caspase-mediated proteolysis of PARP-1, which is cleaved into 24- and 89-kDa peptides [24]. This last fragment retains basal, but not DNA damage-sensitive, activity [25]. Caspase 3 is one of the PARP-1 inhibitory caspases activated by proteolytic cleavage during the early stage of apoptosis [26]. The cleaved form of Caspase-3 and the 89-kDa PARP cleavage fragment are considered as markers of apoptosis, consequently their measurement is widely used to determine the apoptosis rate [27]. As expected, in prolonged in vitro stimulation we detected an increase in the percentage of active Caspase-3 and cleaved PARP-1 positive cells.

These experiments show that flow cytometry is a feasible approach in tracking cell responsiveness to an inflammatory stimulus by monitoring the level of intracellular PAR.

We then used this assay to evaluate in vivo PARP-1 activation in bovine milk leukocyte subpopulations from naturally infected dairy cows. Specifically, a flow cytometric assay was set up to evaluate the levels of PAR, as indicators of PARP activity in specific somatic cell subsets in composite milk samples. The results show that the levels of PAR increased in the lymphocyte and macrophage populations during intramammary infection.

This evidence indicates that PARP-enzymes are active in these cells during bacterial infection. It is notable that we did not find any decrease in PAR level linked to cell death in milk samples, as shown in the prolonged in vitro LPS treatment. This result may depend on the dynamics of the immune response, where necrotic/apoptotic immune cells are continuously replaced by new cells from the bloodstream to fight infection.

Furthermore, the PAR level correlated positively with the SCC and hence with the inflammatory udder status, also highlighting a different contribution of each cellular subset.

## 5. Conclusions

In conclusion, this study shows, for the first time, that flow cytometry can be usefully employed to evaluate PARP activation, including in in vivo milk leukocytes subpopulations from naturally infected dairy cows. Although further research is required to clarify the underlying mechanisms, the current study corroborates the involvement of the PARylation process in intramammary infection.

This finding has significant translational implications: (1) flow cytometric measurements of PAR levels could be used to easily detect and follow the course of different diseases associated with the altered synthesis of PAR, including autoimmune and inflammatory diseases [28]; (2) flow cytometry-based PAR detection may be useful to evaluate the potential therapeutic effect of the pharmacological inactivation of PARP during the clinical management of disease; (3) bovine mastitis may represent a good model to study the PARP role during inflammatory diseases, opening new perspective to the “One Health” approach.

## 6. Patents

The described flow cytometric technique to detect poly(ADP-ribose) (PAR) in milk is covered the Italian patent application (Number 102017000100555).

## Figures and Tables

**Table 1:** Descriptive statistics of milk somatic cells (SCC).

Status	N	SCC (Cells/mL)		*p*-Value
		Mean	SE	
Not Infected	9	109,000	66,672	0.009 *
Infected	10	254,000	153,323	

* Unpaired Student’s *t*-test.

**Table 2:** Correlations between SCC and PAR level in milk leukocyte subpopulations.

		SCC Cells/mL	Lymphocytes (PAR MFI)	Macrophages (PAR MFI)	Neutrophils (PAR MFI)
SCC (cells/mL)	r	1	0.426	0.464	−0.033
	*p*-value		0.069	0.045	0.894
Lymphocytes (PAR MFI)	r		1	0.523	0.227
	*p*-value			0.022	0.351
Macrophages (PAR MFI)	r			1	0.448
	*p*-value				0.054
Neutrophils (PAR MFI)	r				1
	*p*-value				

## Data Availability

The data presented in this study are available on request from the corresponding author.

## Supplementary Materials

The following are available online at https://www.mdpi.com/2073-4409/10/3/599/s1, Figure S1: Flow cytometry and western blotting analysis of PAR levels in PBMC treated with LPS and Doxorubicin in combination with the PARP inhibitor ABT-888, Figure S2: Western blotting analysis of PAR, PARP1 and cleaved Caspase -3 in PBMC treated with LPS for 16 h, Figure S3: Western blotting analysis of PAR levels in milk cells from infected/uninfected animals.
