# Supplementary material for: New Insights into the Significance of PARP-1 Activation: Flow Cytometric Detection of Poly(ADP-Ribose) as a Marker of Bovine Intramammary Infection
Source: Cells. 2021 Mar 9;10(3):599. doi: 10.3390/cells10030599 (PMC8001672; doi:10.3390/cells10030599)
Supplement: Supplementary file 1 [file cells-10-00599-s001.pdf]

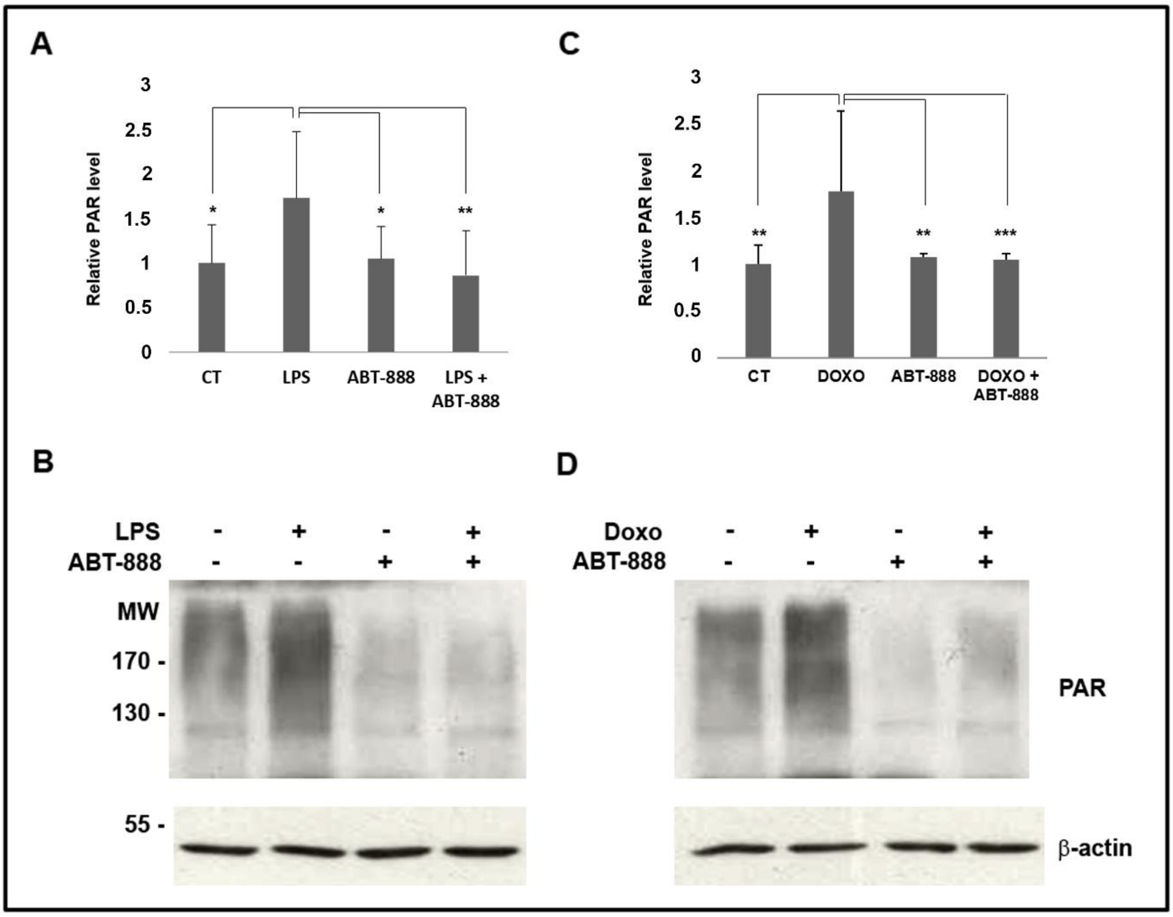

Figure S1. Flow cytometry and western blotting analysis of PAR levels in PBMC treated with LPS and Doxorubicin in combination with the PARP inhibitor ABT-888. Flow cytometry results (**A**) and Western blotting (**B**) analyses of PAR levels in PBMC treated with/without LPS (1 μg/mL) and ABT-888 (1 μM) for 1 hour. Flow cytometry results (**C**) and Western blotting (**D**) analyses of PAR levels in PBMC treated with/without Doxorubicin (DOXO, 1 μM) and ABT-888 (1 μM) for 2 hours. Data in (**A**) and (**C**) are mean ± SD of three experiments using PBMC from three different animals and indicate the fold change relative to the PAR level in the control group. Pairwise comparisons were performed by one-way ANOVA with post-hoc Bonferroni correction. * $p \leq 0.05$, ** $p \leq 0.01$, *** $p \leq 0.001$. Images in (**B**) and (**D**) show representative blots from one animal. β-actin was used as a loading control in Western blotting experiments.

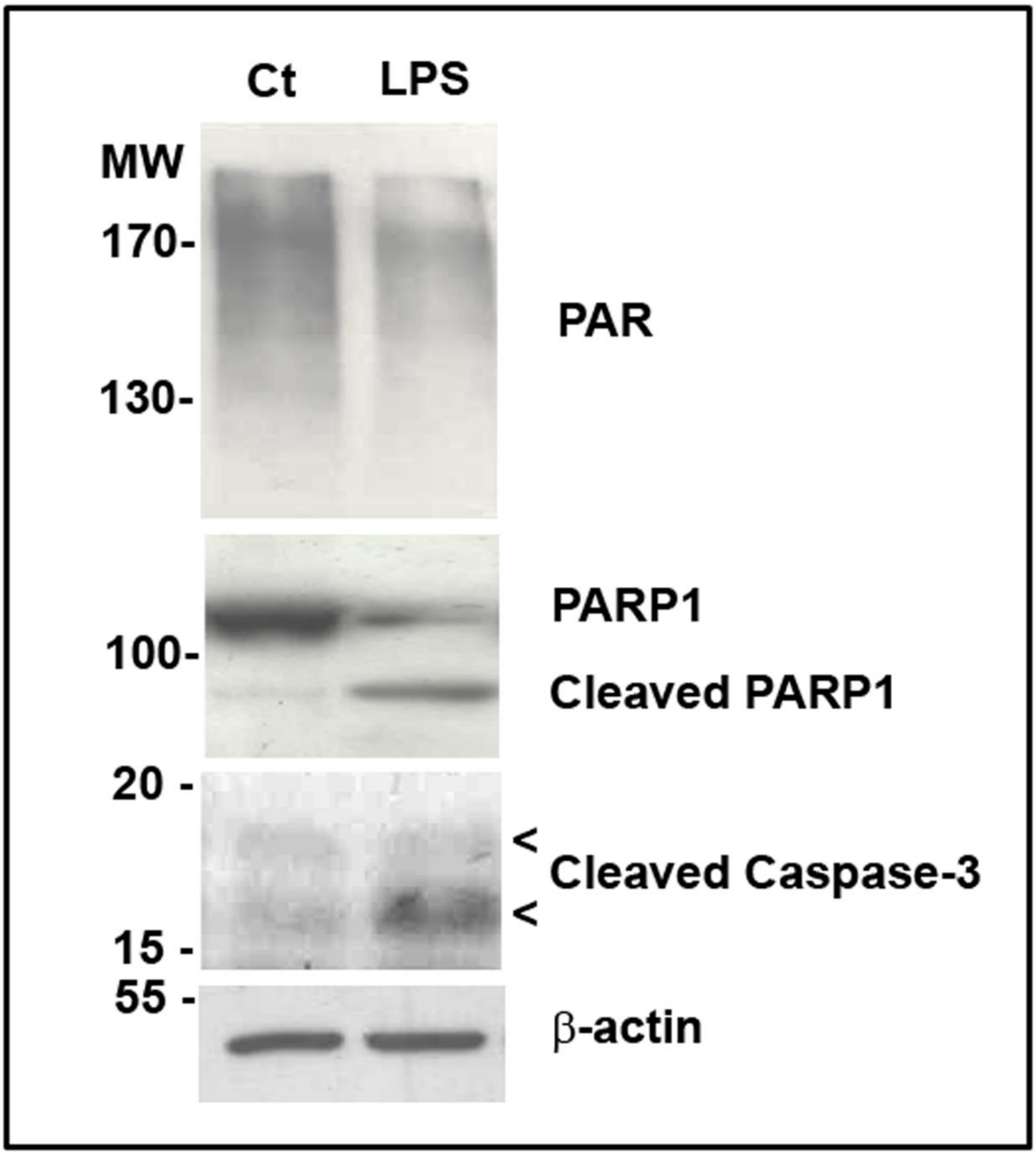

Figure S2. Western blotting analysis of PAR, PARP1 and cleaved Caspase -3 in PBMC treated with LPS for 16 hours. Western blotting analysis of PAR, PARP1 and cleaved Caspase -3 in PBMC treated with/without LPS (1 μg/mL). The image shows a representative blot from one animal. β-actin was used as a loading control.

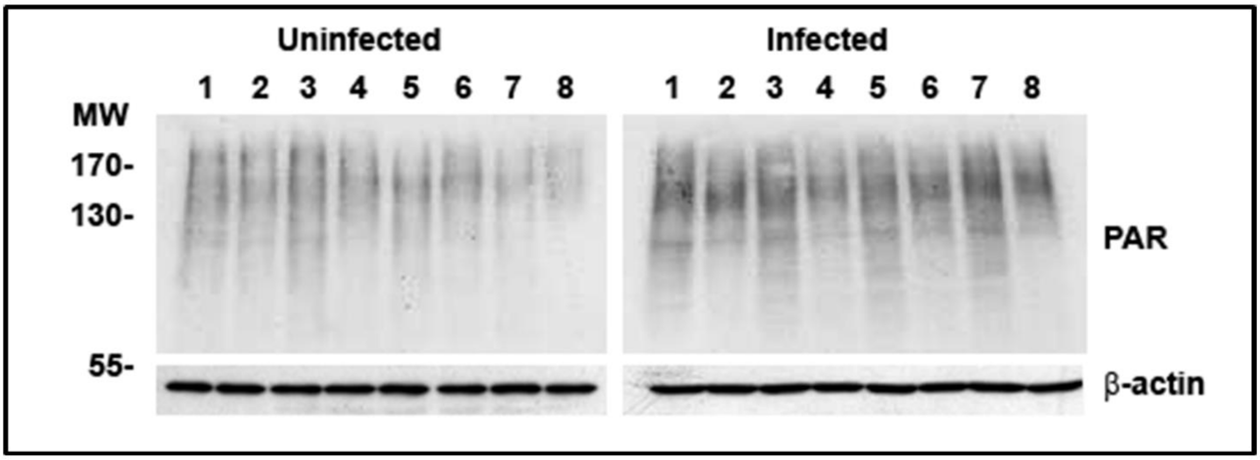

Figure S3. Western blotting analysis of PAR levels in milk cells from infected/uninfected animals. The Image shows a representative blot from eight infected and uninfected cows. β-actin was used as a loading control.
